# Graphene and Iron Reinforced Polymer Composite Electromagnetic Shielding Applications: A Review

**DOI:** 10.3390/polym13152580

**Published:** 2021-08-03

**Authors:** Saba Ayub, Beh Hoe Guan, Faiz Ahmad, Yusuff Afeez Oluwatobi, Zaib Un Nisa, Muhammad Faisal Javed, Amir Mosavi

**Affiliations:** 1 Department of Fundamental and Applied Sciences, Universiti Teknologi PETRONAS, Bandar Seri Iskandar 32610, Perak, Malaysia; beh.hoeguan@utp.edu.my (B.H.G.); oluwatobi_20001848@utp.edu.my (Y.A.O.); zaib_20001001@utp.edu.my (Z.U.N.); 2 Department of Mechanical Engineering, Universiti Teknologi PETRONAS, Bandar Seri Iskandar 32610, Perak, Malaysia; faizahmad@utp.edu.my; 3 Department of Civil Engineering, COMSATS University Islamabad Abbottabad Campus, Abbottabad 22060, Pakistan; arbabfaisal@cuiatd.edu.pk; 4 Faculty of Civil Engineering, Technische Universität Dresden, 01069 Dresden, Germany; 5 John von Neumann Faculty of Informatics, Obuda University, 1034 Budapest, Hungary; 6 Information Systems, University of Siegen, 57072 Siegen, Germany; 7 Department of Informatics, J. Selye University, 94501 Komarno, Slovakia

**Keywords:** electromagnetic inference, shielding effectiveness, graphene, iron, polymer, composite materials, materials, review, materials design, computational materials design

## Abstract

With advancements in the automated industry, electromagnetic inferences (EMI) have been increasing over time, causing major distress among the end-users and affecting electronic appliances. The issue is not new and major work has been done, but unfortunately, the issue has not been fully eliminated. Therefore, this review intends to evaluate the previous carried-out studies on electromagnetic shielding materials with the combination of Graphene@Iron, Graphene@Polymer, Iron@Polymer and Graphene@Iron@Polymer composites in X-band frequency range and above to deal with EMI. VOSviewer was also used to perform the keyword analysis which shows how the studies are interconnected. Based on the carried-out review it was observed that the most preferable materials to deal with EMI are polymer-based composites which showed remarkable results. It is because the polymers are flexible and provide better bonding with other materials. Polydimethylsiloxane (PDMS), polyaniline (PANI), polymethyl methacrylate (PMMA) and polyvinylidene fluoride (PVDF) are effective in the X-band frequency range, and PDMS, epoxy, PVDF and PANI provide good shielding effectiveness above the X-band frequency range. However, still, many new combinations need to be examined as mostly the shielding effectiveness was achieved within the X-band frequency range where much work is required in the higher frequency range.

## 1. Introduction

**Figure 1:**
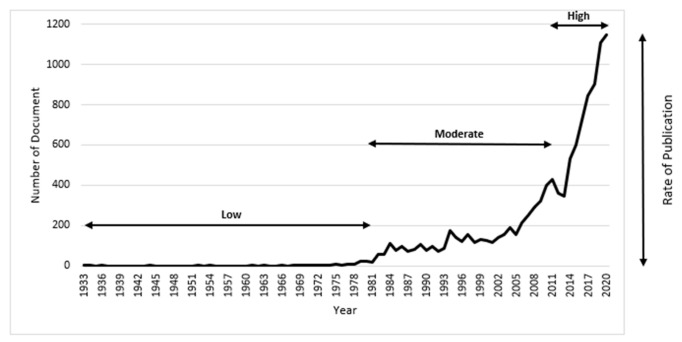
Studies conducted on EMI shielding.

Electromagnetic interference (EMI) has emerged as a global issue due to the rapid growth of electronic devices and their usage in day-to-day life [1]. Electromagnetic pollution is triggering loss of data, signal disturbing, system failures and most importantly causing a serious threat to information communication security and human health [2,3,4]. Owing to the increased usage of these electromagnetic devices, radiation of the electromagnetic (EM) wave has become a serious concern because these radiations not only become disastrous for electronic equipment but also affecting human health [5]. With the advancement in technologies, human exposure to electromagnetic fields is getting common and unavoidable [6,7,8]. Electromagnetic waves do not deflect by any magnetic or electric field and cause serious issues [9]. Electromagnetic waves are causing damages in various forms such as changes in physiological indices, genetic effects, health, and immune functions. With time, the adverse effects are getting higher which requires vital attention [10,11]. The penetration of the EM waves in the shielding materials is a critical aspect at higher frequencies [12]. Electromagnetic waves are non-mechanical which travel at the speed of light. They can be produced by accelerated charge and do not involve any medium for transmission [9]. Electromagnetic waves are also known as electromagnetic radiation as they radiate from charged electrical particles. The transmission could be through air, space, or any other substance. Low frequency electromagnetic waves are stated as electromagnetic fields, whereas high frequency electromagnetic waves are known as electromagnetic radiations [13,14]. EMI shielding has been in consideration since World War II to reduce the impact of electromagnetic waves on electronic appliances [15]. As per Scopus [16] database, Figure 1 portrays the number of experimental studies conducted on EMI shielding to date.

From Figure 1, it can be observed that work started on electromagnetic shielding in 1933. The studies conducted on electromagnetic shielding were fewer from 1933 to 1981 and started to rise later. The peak was observed in 2020 with 1146 publications. Over time, a gradual increase can also be observed, which indicates the importance of this issue. Many researchers have introduced various materials to overcome the EMI effect by providing better electromagnetic shielding effectiveness. Some pre-existing materials with hidden shielding properties were identified, and some were made in labs and later were implemented for industrial usage. The most common materials used as electromagnetic shielding are metals, carbon, iron, graphene and polymers, etc. Various EMI shielding materials have been developed and implemented in industry to tackle electromagnetic inference. Researchers have adopted different strategies to deal with the EMI issue where some used material coatings and some developed new composites. The preferences of selection of materials have changed as their properties have been explored more in-depth. In the mid-1900s different materials were introduced as EMI shielding materials. Nickel-based composites were famous as coatings to enhance EMI shielding. Besides that, copper, silver and graphite coatings were also utilized as they form a good barrier to protect devices from ambient electromagnetic interference [17]. Macfarlane et al. [18] conducted a study, where an yttrium barium cuprate superconductor was used as an electromagnetic shielding material. However, it is universal that all the superconductive composites give better EMI shielding. YBa_2_Cu_3_O_7−x_ superconductive material was used for EMI shielding, where it was revealed that the composite does not provide effectiveness against electromagnetic waves [19]. Carbon fibre was also used as EMI shielding, where it was reported that with an increase in the fibre content, the conductivity increases, which enhances the EMI shielding effectiveness [20,21].

Metals have received attention as EMI shielding materials when used individually and in combination with other materials by forming alloy composites. Moreover, metal fillers were also used for enhancing EMI shielding effectiveness [22,23,24,25,26]. The most used materials are metal sheets and metal foam. Metal sheets include brass, silver, copper, nickel, tin, and steel. The effectiveness of electromagnetic shielding gets affected by the metal’s physical properties such as thickness, weight, permeability, conductivity and solderability, which change the reflection and absorption capabilities. Metals’ properties play an important role in the material section as EMI shielding. High conductive metals (brass, copper, silver) reflects electrically dominant waves, whereas, less conductive metal (steel) absorb magnetically dominant waves [27,28]. Metal foam got hype due to its use in both scientific and industrial applications. Metal foams are composite structures comprised of metal (aluminium) and gas (mainly air is added). The combination of these two results in a disordered wire mesh having low density [29,30,31,32,33]. Authors earlier gave a brief state of the art review on graphene and iron reinforced polymer composites for electromagnetic shielding applications [28]. However, still there is a gap for an in-dept understanding and comprehensive review. Carbon materials and their composites gained attention in the field of EMI shielding because of their better conductivity and flexibility [34,35,36]. Carbon fillers such as carbon black and carbon fibre were also used to achieve EMI shielding effectiveness. Later on, carbon particles and carbon nanotubes were introduced which showed good results [37]. However, a few studies have suggested that carbon-based materials have limited mechanical flexibility, where metal-based materials suffer from corrosion and are heavy in weight, due to which it becomes difficult to achieve high shielding effectiveness [38]. Therefore, there is a great need for developing a shielding material that is light and durable, at low cost, produces no pollution, have comprehensive performance and shielding frequency bandwidth [39]. Thus, the microstructure of the nanomaterials, the structure of the shield and the inclusion of foreign materials such as materials with dielectric or magnetic dipoles play an important role in the absorption of the EM waves [12].

Conducting polymer composites earned recognition in comparison to metal-based composites due to their flexibility, lightweight and resistance to corrosion [40,41,42,43]. Various researchers utilized polymer-based materials as they are lightweight in comparison to metal-based materials. However, polymer-based materials give less shielding against EMI as they are less conductive and have been used in combination with other materials which comes as a good absorber for electromagnetic waves [44,45,46,47,48]. The polymer-based materials have been identified as the ideal materials for EMI shielding effectiveness [49,50]. With the introduction of the 5th Generation (5G) telecommunication system and high frequency range electronic interfaces, the EM pollution has been increased drastically, hence, the coupling effect of EM radiation and signals interferences requires suppressed [51,52]. Therefore, this study aims to review the past studies conducted to deal with the electromagnetic inferences through graphene, iron and polymer composites. The focus of this review is mainly limited to the combination of the Graphene@Iron@Polymer family as an EMI shielding material within the range of X-band frequency and above. The study will provide a benchmark for future researchers to select the right material combination for EMI shielding effectiveness.

## 2. Scope of Review

As the purpose of this study is to review the past literature of electromagnetic inferences (EMI) shielding materials, therefore, only those articles which were available with the combination of Graphene@Iron, Graphene@Polymer, Iron@Polymer and Graphene@Iron@Polymer composites were considered. For metals, articles where these were combined with graphene were also considered. The articles were taken from all the available databases without applying any year limitation. Based on the selected articles keywords analysis was also performed via VOSviewer which shows the relationship and interconnectivity of the articles. Making a detailed review, a gap is also highlighted to guide in the field of EMI shielding materials.

## 3. Electromagnetic Inferences Theory

The practice of blocking conducting or radiating electromagnetic waves into sensitive areas is known as EMI shielding [12,53]. EMI shielding materials have a wide range of applications including commercial and scientific electronics, antenna systems, space explorations, satellite communication, automotive radar, millimetre wave wireless LAN and medical devices [12,54]. EMI shielding materials also have a wide range of military applications such as stealth in which radar absorbing materials (RAMs) are used to reduce the detectability of the target by cancelling reflections of a radar signal impinging to its surface [12,55,56].

**Figure 2:**
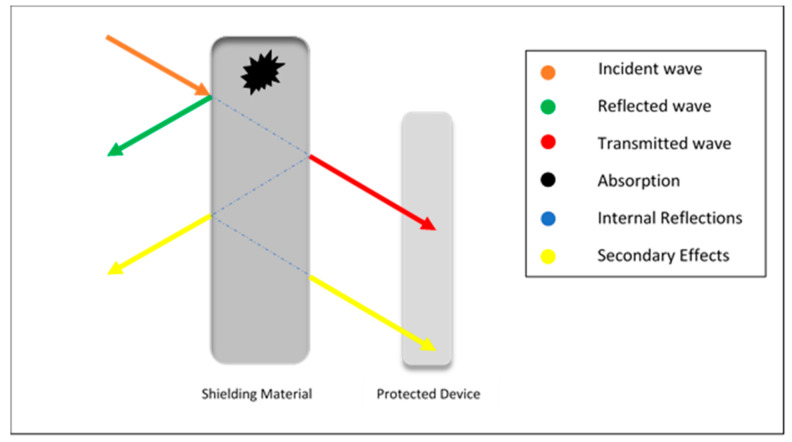
Schematic illustration of electromagnetic wave strike on protected device.

Based on the frequency, electromagnetic waves are classified as ionizing and non-ionizing radiations. Ionizing radiations are high frequency electromagnetic waves that include X-rays and gamma rays, whereas non-ionizing radiations are low frequency electromagnetic waves that include microwaves fields, infrared radiation, ultraviolet radiation and radiofrequency [57]. When electromagnetic waves strike the EMI shield, the occurrence can be a reflection, absorption, transmission, and multiple reflections [58,59,60,61,62] as shown in Figure 2.

Reflection is considered as a primary shielding mechanism in a homogeneous conductive material, where materials must possess mobile charge carriers to interact with EM waves [58,60,62]. Absorption is considered the second most essential mechanism which depends on the shielding thickness. Absorption capacity increases when the material acquires electrical and magnetic dipoles in contact with EM waves. Multiple reflections are the third shielding mechanism which usually decreases if the shield is thinner than the skin depth but could be ignored if it is thicker than the skin depth. When an EM wave passes a conductive material, its strength decreases [48]. Multiple reflections can be secondary reflection and secondary transmission [63]. Eliminating the secondary microwave pollution generated by a strong reflection of electromagnetic shielding material is also important which can be achieved by developing the high efficiency electromagnetic wave absorbing material [64]. The overall shielding effectiveness for a material can be calculated by using Equation (1) [65]:

SE_overall_ = SE_R_ + SE_A_ + SE_MR_ (1)

where, SE = shielding effectiveness, R = reflection, A = absorption and MR = multiple reflections

SE_R_ is the reflection loss that is linked with impedance mismatching among EM waves and shielding material. Reflection loss can be calculated by using Equation (2) [66]:

(2) $${\mathrm{SE}}_{R}=20\log\left|\frac{Z_{\mathrm{in}}-1}{Z_{\mathrm{in}}+1}\right|$$

where, Z_in_ is the impedance of the absorber.

SE_R_ is a function of permeability and conductivity which decreases with frequency. EM waves amplitude decreases upon passing through the material where absorption loss occurs. The absorption loss occurs due to material heating and ohmic losses when current is induced. It can be calculated by using Equation (3) [65]:

(3) $${\mathrm{SE}}_{A}=20\log e^{\frac{d}{\delta }}\;$$

(4) $$\delta =\sqrt{\frac{2}{\omega \mu \sigma }}$$

where, d = material thickness, δ = skin depth, μ = permeability, σ = conductivity, ω = wave frequency.

Upon passing an EM wave through the material, the intensity reduces, which is known as the attenuation constant. SE_A_ depends on thickness, permeability, and conductivity. The dependency of both SE_R_ and SE_A_ on conductivity and permeability shows that the shielding is controlled by absorption rather than the reflection in conducting metals. Besides that, permittivity is also important for enhancing the SE_R_ and SE_A_ [65].

SE_MR_ (multiple reflections) can be calculated by using Equation (4) [65]:

(5) $${\mathrm{SE}}_{\mathrm{MR}}=20\log\left(1-e^{\frac{-2d}{\delta }}\right)$$

SE_MR_ depends on the thickness which is tightly linked with absorption and plays a vital role for definite geometries and porous structures. The EMI performance increases in hollow porous structures due to their unique properties such as permeability, low density, tailorable internal structures and high surface area. When shielding material thickness is greater than penetration depth, multiple reflections can be ignored [65]. The presence of mobile charges primarily causes the reflection by the conductors. The reflection mechanism stimulates the secondary EMI pollution, that is why the focus of the shielding materials is to provide a strong EMI absorption [67]. The EMI absorption mechanism of a material is categorized as: (i) magnetic loss (occurs due to permeability), and (ii) dielectric loss (occurs due to permittivity) [68]. Regrettably, most of the polymer composites suffer from low dielectric loss [52,68,69,70]. Even though in materials heat was dissipated as the absorbed EM radiation, still, the heating effect is not taken into the consideration [71,72,73].

## 4. Graphene-Based Composites

**Figure 3:**
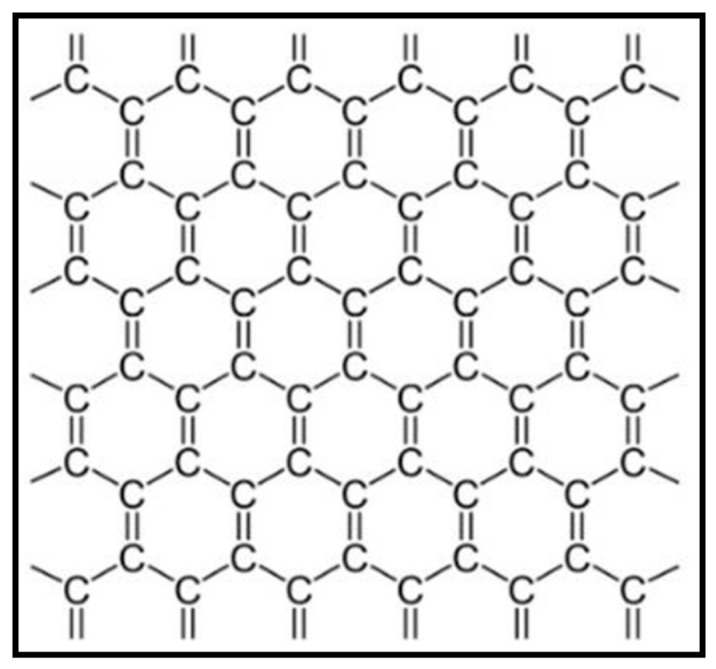
Graphene structure.

Graphene is a 2D planar sheet and is an allotrope of carbon that is organized into a hexagonal lattice as shown in Figure 3. A single graphene sheet has a honeycomb structure, which forms due to the arrangement of a single layer of carbon atoms. When several sheets pile on each other, they form multi-layer graphene. The structure of graphene is such that each carbon atom is attached to the other three carbon atoms which provide better stability and high tensile strength [74,75].

**Figure 4:**
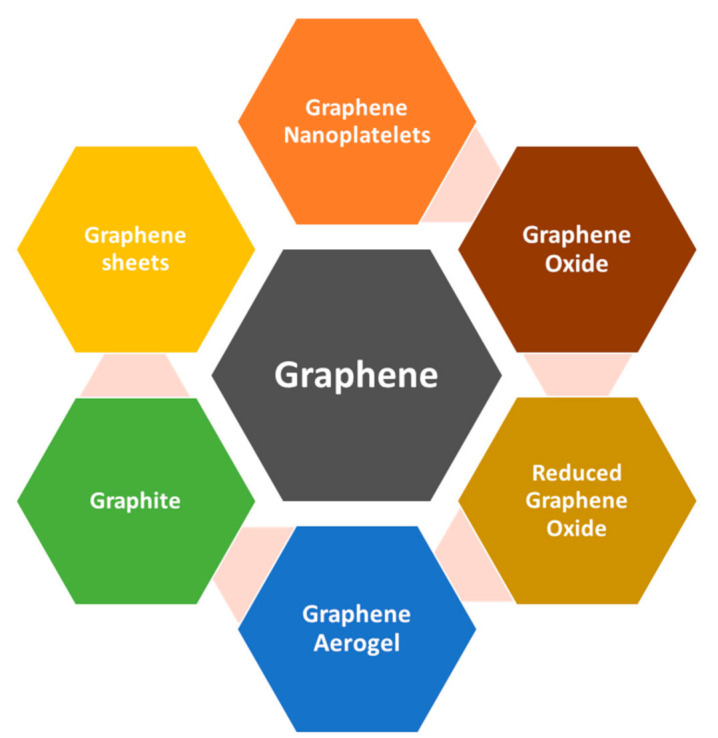
Graphene family for EMI shielding.

Technically, it is a non-metal material but it is deemed a quasi-metal because it displays semi-conducting metal properties. Graphene possesses unique properties which do not exist in other non-metallic materials, therefore making it superlative material for electronic applications usage. The single-layer graphene sheet has 1.0 TPa Young’s modulus and can bear stress up to 42 Nm^−1^, that is why it is considered as one of the strongest materials available [76]. The electron mobility of graphene in comparison to silicon is 100 times faster and it conducts twice as much heat as diamond. The electrical conductivity of graphene in comparison to copper is 13 times better [77]. The graphene family that has been used as EMI shielding material are presented in Figure 4. The types of graphene are distinguished based on their structure. The modification has been brought into the material by altering the structural properties.

**Figure 5:**
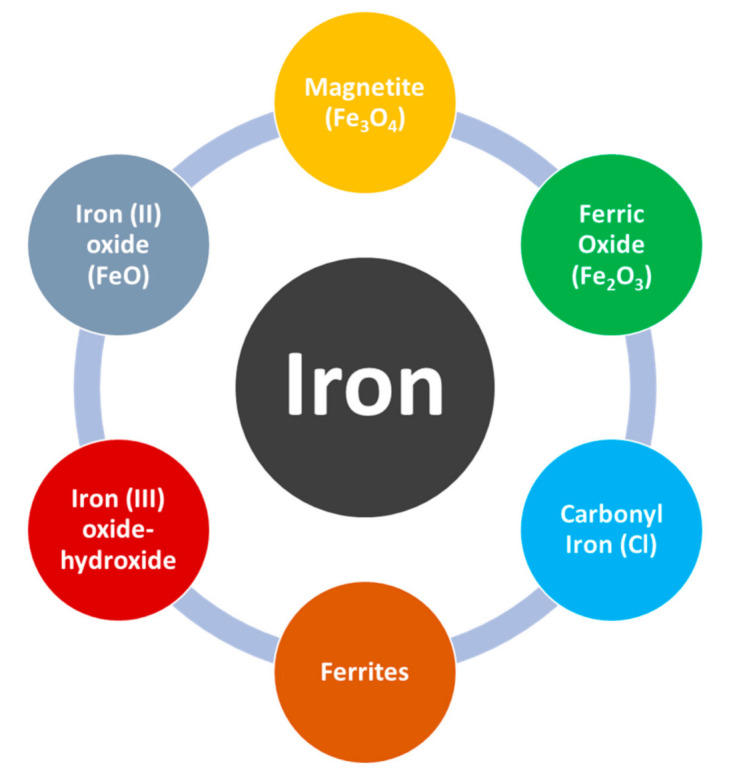
Iron component types.

Among metals, iron has gained much popularity due to its magnificent properties to deal with EMI. In recent years magnetic nanostructures are in demand as they provide good absorption when combined with graphene. In comparison to other metals for EMI shielding applications, iron is the most desirable material due to its high natural availability, low facile synthesis cost and high biocompatibility and biodegradability nature [65]. The various types of iron which have been used extensively in the field of EMI are presented in Figure 5. which is adapted from [65].

### 4.1. Graphene@Iron Composites

#### 4.1.1. Keywords Analysis of Graphene@Iron Composites Articles

**Figure 6:**
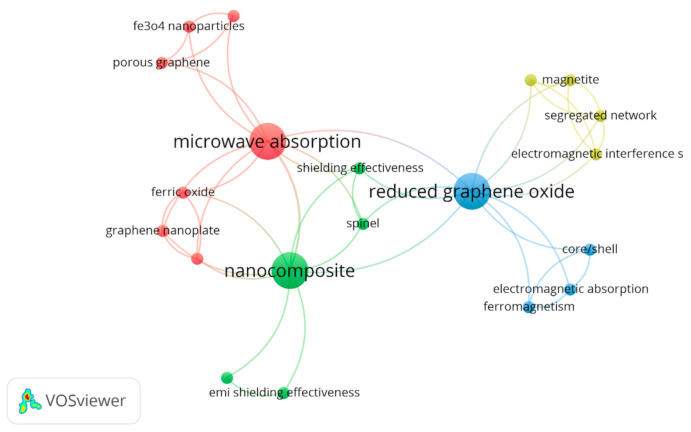
Keyword Analysis of Graphene@Iron-based composites articles.

Keywords analysis is important in evaluating the area of interest in the articles. It helps to identify the differences and research growth in a particular studied area. Moreover, the co-occurrence analysis of keywords shows the relationship build due to various keywords. Based on the selected articles of Graphene@Iron composites, keyword analysis of the selected articles was made by VOSviewer where the mapping is shown in Figure 6.

The first cluster with red nodes was assembled across the term “microwave absorption” having a maximum occurrence of 3. In the same cluster, “Fe_3_O_4_ nanoparticles”, “ferric oxide”, “graphene nanoplate”, “hybrid material”, “impedance matching”, “microwave absorption”, “porous graphene” exists with just one occurrence each. The following cluster demonstrates the interest of researchers in microwave absorption of the included composites.

Similarly, the green cluster around the keyword “nanocomposite” having a maximum cooccurrence of 3. The same cluster consists of keywords “EMI shielding effectiveness”, “Fe_3_O_4_ @gnp hybrid”, “nanocomposite”, “shielding effectiveness” and “spinel”. Likewise, the third cluster is blue coloured with the keyword “reduced graphene oxide” with a maximum occurrence of 3. While the last yellow cluster is closely linked with the blue cluster having keywords “electromagnetic interference shielding”, “magnetite”, “natural rubber” and “segregated network” with 1 occurrence each.

#### 4.1.2. Interpretation of Graphene@Iron Composites Articles

Different types of graphene such as pristine graphene, reduced graphene oxide, and graphene oxide have been investigated for EMI shielding alone and at times as a combination with other materials which possess conductive and magnetic properties [46,78,79,80]. Being a 2D nanomaterial, graphene has remarkable electrical properties that is why it has been used extensively for EMI shielding. With iron and graphene combinations researchers introduce a third material to enhance their properties such as epoxy, polymer, silicon dioxide (SiO_2_), titanium dioxide (TiO_2_) etc. The work is still going on with different formed combination to deliver quality results.

Wang et al. [81] used a hydrothermal method to fabricate hollow ZnFe_2_O_4_ microspheres@graphene which was decorated with TiO_2_ nanosheets. The highest reflection loss of ZnFe_2_O_4_@graphene@TiO_2_ with the coating of 2.5 mm was up to −55.6 dB at 3.8 GHz, where the absorbing bandwidth surpassing −10 dB at 6.4 GHz with the same thickness. The results prove that ZnFe_2_O_4_@graphene@TiO_2_ provides good absorption in low frequency. Mederos-Henry et al. [82] conducted a study on low frequency microwaves using the Pechini sol-gel method, where a new microwave absorber material was synthesized having the combination of reduced graphene oxide which was covered with Fe@_ϒ_-Fe_2_O_3_ and Fe/Co/Ni. It was revealed that the microwave absorption efficiency (0.4 MHz–20 GHz) comes in the range of 60%–100% by using these materials, depending on the metallic particles’ nature grafted on reduced graphene oxide. Chen et al. [83] adopted a scalable coprecipitation process to form aerogels exhibiting strong electromagnetic wave absorption material using cellulose/reduced graphene oxide and Fe_3_O_4_ with the loading of 8 wt.% and 15 wt.%. With the aerogel thickness of 0.5 mm, 32.4–40.1 dB EMI shielding effectiveness was achieved for 8.2–12.4 GHz frequency. The shielding effectiveness got higher by introducing a larger amount of reduced graphene oxide with loading varies between 3–8 wt.% and increasing the thickness between 0.5–2 mm. Shielding effectiveness reached 49.4–52.4 dB with 2 mm sample thickness. It was concluded that high shielding effectiveness can be achieved with the help of lightweight aerogels.

Kumar et al. [84] utilized a solvothermal method to synthesize the NiFe_2_O_4_ nanoparticles with reduced graphene oxide to observed EMI shielding performance within the frequency range of 8.2–12.4 GHz. Significant dielectric and magnetic loss were shown by the nanocomposite compared to RGO with improvement in electromagnetic wave absorption. With 2 mm thickness, the shielding effectiveness of 38.2 dB at 10.8 GHz was achieved with a 35/65 ratio of NiFe_2_O_4_/RGO. Prasad et al. [85] decorated magnetic CoFe₂O₄ nanoparticles on MoS₂-reduced graphene oxide surface using hydrothermal method. The EMI shielding effectiveness was examined within the range of 8.0–12.0 GHz, where the pure MoS₂-RGO nanocomposite gives shielding effectiveness of 16.52 dB while the MoS₂-RGO/CoFe₂O₄ nanocomposite provides shielding effectiveness of 19.26 dB.

Jiang et al. [64] used a facile solvothermal method for developing a magnetic Fe_3_O_4_ combined with graphene nanoplates by constructing spherical Fe_3_O_4_ particles with integrity crystal on the graphene sheet surface. The results showed a better absorption performance due to the impedance matching ability of Fe_3_O_4_@f-GNPs compared to dielectric f-GNPs and magnetic Fe_3_O_4_, with a reflection loss of −25 dB at 10 GHz frequency when having 2 mm sample thickness and 2.4 GHz (below −10 dB) effective absorption bandwidth. Moreover, the absorption and total efficiencies were 32 dB and 25 dB respectively, when the Fe_3_O_4_@f-GNPs exhibited shielding efficiency properties with 232 nm Fe_3_O_4_ in the X band. Bhaskaran et al. [86] investigated the EMI shielding effectiveness of epoxy nanocomposites containing Fe_3_O_4_ nanoparticles coated graphene nanoplatelets, where by using a co-precipitation technique and a solvent-less approach, hybrid nanostructures were synthesized in situ. The results showed the high EMI shielding effectiveness of Fe_3_O_4_@GNP compared to other samples having equivalent loading of GNP and Fe_3_O_4_. The sample containing a 1:3 ratio of Fe_3_O_4_:GNP hybrid with 1 mm thickness reduced incident wave power up to 89% with EMI shielding effectiveness of 9.6 dB. Fei et al. [87] fabricated a multilayer sandwich structure from graphene nanoplates (GNPs), ferric metal-organic frameworks (MOFs) (MIL-88B)-derived magnetic carbon-based materials (C-MIL-88B) via a filtration assisted self-assembly method. With the insertion of Fe_3_O_4_-C, C-MIL-88B/GNP adequate results in terms of magnetization and conductivity were shown, where the composite film consisting of five layers with the thickness of 0.12 mm showed effective EMI shielding with the value of 28 dB in the X-band frequency range with 86% of power coefficient absorption. Zheng et al. [88] synthesized porous graphene (PG) with Fe_3_O_4_ via in situ growth. It was revealed that PG-Fe_3_O_4_ provides excellent microwave absorption where the reflection loss came as −53 dB at 5.4 GHz frequency. PG which is formed by structure modification is important in achieving the results and showed better performance when get compared with the ordinary graphene.

Graphene oxide (GO) was synthesized with carbonyl iron particles (CIP) by the wet stirring process. The shielding effectiveness of the composite was examined within the range of 0–18 GHz where the maximum reflection loss of −56.4 dB was achieved while keeping the thickness as 1.9 mm. The composite is proposed to be used in X-band as well as Ku-band [89]. Ag@Fe_3_O_4_ was synthesized with reduced graphene oxide by using the solvothermal method. In the testing range (2–18 GHz) the maximum reflection loss came as −40.05 dB at 11.9 GHz [90]. In another study, Fe_3_O_4_@C/RGO was synthesized using the solvothermal method where the reflection loss of −59.23 dB was achieved at 6.24 GHz within the frequency range of 2–18 GHz while keeping the thickness at 3.57 mm [91]. Zhang et al. [92] synthesized natural rubber with magnetic iron oxide and reduced graphene oxide, forming an NRMG composite. The shielding effectiveness of 26.4 dB was achieved within the testing range of X-band while keeping the sample thickness as 1.6 mm. Zhang et al. [92] synthesized graphene and Fe_3_O_4_ with carbon (C) nanoparticles to form a shielding composite. The reflection loss of −55.05 dB was achieved within the 2–18 GHz frequency range. Liu et al. [93] synthesized magnetic graphene (G) with Fe_3_O_4_ (F) hybrid material via the hydrothermal method. The shielding effectiveness was tested within the 2–18 GHz frequency range. While keeping the sample thickness as 1.9 mm, shielding effectiveness of 20 dB was achieved. Yin et al. [94] Ni_0.5_Co_0.5_Fe_2_O_4_/graphene via a hydrothermal method to form a shielding composite, where it was tested within the range of 0.58–1.19 GHz. A reflection loss of −30.92 dB was achieved at 0.84 GHz with 4 mm thickness. Guo et al. [95] utilized a vacuum-assisted filtration method to form a shielding composite of RGO/CNF@Ag-Fe_3_O_4_, where shielding effectiveness of 21 dB was achieved in X-band frequency range with 0.11 mm sample thickness. Table 1 shows a summary of graphene and iron-based composites.

## 5. Polymer-Based Composites

Metals have extensively been utilized for EMI shielding, however, due to easy corrosion and difficult processing, their use has been limited [96,97]. Therefore, researchers turned their interest towards an alternative material i.e., polymer-based composites, which are more promising as they are lightweight, having a low cost, more processability and give better performance as compared to metal-based composites [98,99]. Polymer-conductive nanoparticle composite has unique porous morphology which has shown better results as electromagnetic waves absorber. Air insertion in the material permits high electromagnetic waves access which expands interactions with various air-filled pores and high conductive cell walls. As a result, effective electromagnetic wave dissipation occurred within a lightweight structure. It has a massive influence on final shielding properties however the relationship between final morphology and foaming conditions differs and not certainly predictable [63]. Polymer-based composite materials provide exceptional benefits in comparison to metals, such as low density, enhanced flexibility, and ease in processing. However, with limited mechanical properties like inadequate electrical conductivity, lower temperature resistance, it is difficult to utilize polymer for shielding applications, especially under extreme temperature [100]. Hence, such a combination should be created which can overcome the deficiencies of polymer against electromagnetic wave shielding in extreme conditions.

### 5.1. Graphene@Iron@Polymer Composites

#### 5.1.1. Keywords Analysis of Graphene@Iron@Polymer Composites Articles

**Figure 7:**
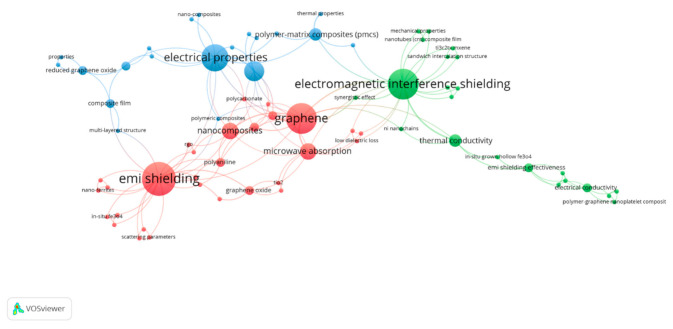
Keywords Analysis of polymer-based composite articles.

Based on the selected articles of Graphene@Iron@Polymer composites, keyword analysis of the selected articles was made by VOSviewer where the mapping is shown in Figure 7.

The first cluster with red nodes was assembled across the term “EMI shielding” and “graphene” having a maximum occurrence of nine and eight, respectively. In the same cluster, the terms “nano composites” and “microwave absorption” with an occurrence of four can be seen. The second cluster with green nodes is representing the second large cluster assembled around the most frequently used term “electromagnetic interference shielding” with the occurrence of eight. This cluster consists of some main words such as: “synergistic effect”, having three occurrences and “EMI shielding effectiveness” with two occurrences. There are also some other keywords relating to the properties such as “magnetic nanoparticles”, “ultrathin film” and “mechanical properties” which shows that the researchers interested in studying the properties of polymers. The third cluster with blue nodes was assembled around “electrical properties” having seven occurrences. This cluster is enriched with many polymers keywords such as “polymers”, “polymer-matrix composites (PMCs)”, “reduced graphene oxide” and many more indicating the study of shielding effect of these polymers and their properties with different parameters.

#### 5.1.2. Interpretation of Graphene@Iron@Polymer Based Composites Articles

Different fillers are used with polymer composites to enhance the underlying matrix material. Carbonaceous fillers such as carbon black, carbon nanotubes, carbon nanofibers, graphene and graphene nanoplates have shown benefits in improving the mechanical properties of polymer composites [101,102]. Adding graphene to a polymer has been shown to result in effective EMI shielding properties as it has the capability to create conductive networks within the polymer matrix [103,104,105]. In addition to improved filler materials, polyaniline (PANI) comes as the matrix material [106]. Shakir et al. [107] evaluated EMI shielding properties by utilizing polymer blends of polyvinyl chloride (PVC) and PANI with graphene nanoplatelets (GNP) insertion. An enhanced electrical conductivity was noticed both for PVC/PANI and PVC/PANI/GNP composites. The EMI shielding effectiveness of 51 dB was achieved in the 18–20 GHz range. Khasim [108] used PANI and graphene nanoplatelet composite for microwave shielding applications. The composite was prepared keeping 1.5 mm thickness by using in-situ polymerization. It was revealed that with 10 wt.% loading of graphene nanoplatelet, high shielding effectiveness (up to 95%) was achieved in X-band frequency. Also, it was revealed that the high absorption occurs due to the dominant absorption mechanism. Having improved conductivity, better thermal stability, and excellent EMI shielding properties, the composite is recommended for its application in X-band microwave frequencies. Jia et al. [109] formed TiO_2_/PANI/Graphene oxide (GO) composite via in-situ growth. The reflection loss was examined within the range of 2–18 GHz where the maximum came as −51.74 dB at 9.67 GHz frequency. Liu et al. [110] used in-situ growth and hydrothermal method to synthesize magnetic graphene with PANI and porous TiO_2_ and tested its EMI shielding efficiency within the range of 2–18 GHz. Keeping the sample thickness as 1.5 mm, a reflection loss of −45.4 dB was achieved.

Wang et al. [111] synthesized graphene@Fe_3_O_4_@PANI decorated with WO_3_ particles by using a hydrothermal method and chemical oxidation polymerization. The spherical nanoparticles of Fe_3_O_4_ and WO_3_ having a diameter of 300–500 nm and 50–150 nm were spread in between graphene@PANI layers. The results showed that graphene@Fe_3_O_4_@PANI@WO_3_ gives better electromagnetic wave absorption as compared to graphene@Fe_3_O_4_ and graphene@Fe_3_O_4_@PANI, where maximum achieved absorption was −46.7 dB with a coating thickness of 4 mm. Whereas, the maximum absorbing bandwidth was ≤10 dB of 1.8 GHz (from 12.4 to 14.2 GHz) with a thickness of 1.5 mm. Wang et al. [112] fabricated a graphene@Fe_3_O_4_@SiO_2_@polyaniline composite which gives better reflection loss of −40 dB at 12.5 GHz with 2.5 mm thickness and absorption bandwidth below −10 dB of 5.8 GHz (from 10.5 to 16.5 GHz) when compared with graphene@Fe_3_O_4_. Zhao et al. [113] used the Hummers method to synthesized polyaniline (PANI), graphene oxide (GO) and Fe_3_O_4_ as EMI shielding composite. With a sample thickness of 3.91 mm, a reflection loss of −53.5 dB was observed within the range of the 2–18 GHz frequency.

In another study, PANI composite comprised of graphene and silver nanoparticles were used as EMI shielding material where the shielding effectiveness of 29.33 dB in 0.4–1.6 GHz frequency range was achieved at 5 wt.% loading [114]. Ma et al. [115] formed Fe_3_O_4_/PANI rod/RGO composites to deal with the EMI which was tested under the range of 2–18 GHz where a reflection loss of −33.3 GHz was achieved. In the study, the material thickness was increased from 1 mm to 4 mm where the maximum reflection loss was achieved at 3.5 mm thickness. It proves that by increasing the sample thickness the shielding effectiveness can be increased but up to a certain level. Wang et al. [116] synthesized graphene@NiO@PANI@Ag using hydrothermal and in-situ growth method. The composite material was tested within the range of 2–18 GHz where a reflection loss was achieved as −37.5 dB with 3.5 mm sample thickness. Zhou et al. [117] formed graphene-doped polyaniline (G-PANI) as shielding composite via in-situ growth. The shielding effectiveness of 32.5 dB was achieved within the range of 2–18 GHz with 1.5 mm thickness. Singh et al. [118] formed a new material ℽ-Fe_2_O_3_ and decorated it with RGO and PANI to observe the shielding efficiency of the composite. The composite was formed via chemical oxidation polymerization and in-situ growth and tested within the X-band frequency range. While keeping the sample thickness as 2.5 mm, total shielding effectiveness of 51 dB was achieved. Wang et al. [119] explored Ti_3_C_2_T_x_ MXene shielding properties by making its composite with Fe_3_O_4_ and PANI polymer. The co-precipitation method was used to prepare the composite and was tested within the X-band frequency range. Shielding effectiveness of 58.8 dB was achieved with 12.1 µm sample thickness. Preeti et al. [120] used the citrate precursor method to synthesize RGO, barium ferrite (BF) and PANI to form a shielding composite where shielding effectiveness of 31.1 dB was achieved in the X-band frequency range. Dar et al. [121] synthesized PANI/Li_0.5_Fe_0.5−x_Gd_x_O_4_ via in-situ growth where the composite was tested within the X-band frequency range. Keeping the sample thickness as 0.2 mm, shielding effectiveness of 42 dB was achieved.

Yan et al. [99] evaluated ultra-efficient electromagnetic interference shielding by using reduced graphene oxide and polystyrene. The results showed that with 3.47 vol% of RGO-based polymer composite, 45.1 dB shielding effectiveness was achieved. Shahzad et al. [122] formed two different composites via a hot compressed method. One was segregated RGO with polystyrene (PS) and the other was conventional RGO/PS. The testing was made from 0–20 GHz where the shielding effectiveness of 29.7 dB and 14.2 dB was achieved with 2 mm sample thickness. Nimbalkar et al. [123] formed a composite by optimizing polycarbonate and graphene nanoplatelets (GNP), using the facile solution method, for electromagnetic interference shielding in X-band. Keeping the composite thickness as 1 mm, 35 dB shielding effectiveness was achieved, where, by increasing the thickness up to 2 mm, 47 dB shielding effectiveness was achieved, indicating that the increase in the thickness directly enhances the shielding effectiveness. Hamidinejad et al. [124] examined lightweight high-density polyethylene (HDPE) with graphene nanoplatelets composites which were fabricated using the supercritical fluid and injection moulding process. The shielding effectiveness of 31.6 dB was achieved in K-band. Lu et al. [125] fabricated ethylene propylene diene monomer rubber (EPDM) with graphene nanoplatelets loading to observed EMI shielding effectiveness in X-band and Ku-band. The results showed that with 8 wt.% of GNP, keeping thickness 0.3 mm, 33 dB shielding effectiveness was achieved in X-band, whereas, in Ku-band, 35 dB shielding effectiveness was achieved.

Zdrojek et al. [126] conducted a study on sub-terahertz radiation shielding by using a graphene-based plastic absorber where PDMS polymer was used. It was observed that being lightweight and nonconductive, graphene-based composites can absorb 99.99% of electromagnetic waves, whereas most metal-based composites simply redirect the radiations. Li et al. [127] formed a copper-coated RGO@PDMS polymer composite by the Hummers method, where a shielding effectiveness of 74.2 dB was achieved in the X-band frequency range. Ni et al. [128] synthesized a graphene aerogel (GA) with PDMS polymer, where shielding effectiveness of 60 dB was achieved within the frequency range of 2–18 GHz. In another study conducted by Fang et al. [129], a 3D-graphene network combined with PDMS was used for high performance EMI shielding. With this combination, 6100 S/m electrical conductivity was achieved even with a low graphene loading of 1.2 wt.%. Also, around 40 and 90 dB, EMI shielding effectiveness was attained in the X-band range when the thickness was kept as 0.25 and 0.75 mm. It is noteworthy that with a 1.2 wt.% loading level, a 256% increase was observed in the tensile strength of the composite. Fang et al. [129] formed a composite of in-situ grown hollow Fe_3_O_4_ with graphene foam (GF) and PDMS by using the solvothermal method for high EMI shielding effectiveness. The results showed that 70.37 dB shielding effectiveness was achieved in the X-band frequency. Nguyen et al. [130] worked on multifunctional broadband EMI shielding skins using MXene(Ti_3_C_2_T_X_)/graphene/PDMS composites. MXene is a newly developed shielding material that provides high shielding effectiveness [131]. Fe_3_O_4_ nanoparticles added with Ti_3_C_2_T_X_ was coated on graphene foams, where the thickness was kept as 1 mm. The results revealed that an excellent EMI shielding effectiveness was achieved in X-band with 80 dB, whereas, in Ka-band, 77 dB shielding effectiveness was achieved. Liang et al. [132] optimized flexible polyvinylidene fluoride (PVDF) with high-aligned graphene nanosheets and Ni nanochains for EMI shielding. With sample thickness kept as 0.5 mm in K-band range, 43.3 dB shielding effectiveness was achieved. Whereas, by increasing the thickness up to 0.7 mm, 51.4 dB shielding effectiveness was achieved within the same frequency range. Sharma et al. [133] grow copper sulphide (CuS) flowers on graphene oxide and later mix it with PVDF polymer. The composites showed shielding effectiveness up to −25 dB at the 12–18 GHz frequency range. Multi-layered graphene nanosheets synthesized with Fe_3_O_4_ and PVDF showed better results where shielding effectiveness of 52 dB was achieved at X-band frequency range while keeping the sample thickness as 0.3 mm [134]. With in-situ growth, RGO and hematite nanohybrids were synthesized with the addition of PVDF. While keeping its loading as 5 wt.%, the maximum absorbing value of −43.97 dB was achieved at 5 GHz [135]. Liang et al. [136] synthesized graphene (Gn) and silicon carbide nanowires (SiCnw) with PVDF via electrostatic assembly and solution casting method. Shielding effectiveness of 32.5 dB was achieved with 1.2 mm sample thickness when tested in the X-band frequency range. Sabira et al. [137] synthesized PVDF with graphene nanocomposite via a solution casting method. Shielding effectiveness of 47 dB was achieved within the X-band frequency range with 20 µm thickness. Qi et al. [138] worked on the three-layered sandwich structure of PVDF, graphene nanoplatelets, nickel (Ni) and carbon nanotubes (CNT). The composite was tested for a three-layered and six-layered structure where shielding effectiveness of 41.8 dB and 46.4 dB was achieved at 15 GHz with a fixed thickness of 0.6 mm. Gargama et al. [139] synthesized PVDF with nanocrystalline iron (n-Fe) to form a shielding composite which was tested within the X-band frequency range. The composite provided shielding effectiveness of 40.21 dB with a 1.93 mm thickness sample. PVDF was also synthesized with ferrosoferric oxide decorated polyaniline/single wall carbon nanohorn (PFC) to form a shielding composite. A reflection loss of −29.7 dB appeared within the Ku-band with 2 mm thickness [140].

Liang et al. [132] optimized 3D copper nanowires-thermally annealed graphene aerogel (CuNWs-TAGA) with epoxy by a thermal annealing method. While keeping the loading of CuNWs-TAGA as 7.2 wt.%, shielding effectiveness was achieved up to 47 dB in the X-band frequency range. Wu et al. [141] synthesized RGO modified carbon fibre (RGO-CF) with the addition of epoxy (EP) using chemical reduction and electrophoretic deposition methods. With a thickness of 3–5 mm, the maximum shielding effectiveness of 37.6 dB was achieved within the X-band frequency range. Liu et al. [142] synthesized 3D network porous graphene nanoplatelets (GNP) with Fe_3_O_4_ and epoxy to form a shielding composite. With 7 wt.% loading of GNP and Fe_3_O_4_, 37.03 dB shielding effectiveness was achieved in the X-band frequency range. A three-phase composite (graphite nanoplatelets (GNP)/carbonyl iron (Fe)/epoxy) was fabricated using a sonication method. The shielding effectiveness was evaluated from 1–67 GHz with various thickness and loadings. It was observed that with 5 mm thickness of the sample and 5 wt.% GNP loading, the reflection loss came as −78 dB [143]. Chen et al. [144] optimized thermally reduced graphene oxide (TGO), magnetic carbonyl iron (CI) and epoxy. The composite was tested in the X-band range where shielding effectiveness of 40 dB was achieved at 4 mm thickness.

Wu et al. [145] synthesized graphene carbon filler (GCF), with magnetic graphene (MG) and epoxy (EP) to form a shielding composite where GCF loading was 0.5 wt.% and MG loading was 9 wt.%. The testing range was from 18–26 GHz where shielding effectiveness of 51.1 dB was achieved. Jaiswal et al. [146] synthesized reduced graphene oxide and ferrite nanofiller with epoxy to form a shielding composite. While keeping the epoxy loading as 60 wt.%, a reflection loss of −10.26 dB was achieved with a 3 mm sample thickness in the 2–18 GHz frequency range. Tolvanen et al. [147] synthesized biodegradable multiphase polylactic acid with biochar and graphite using the hot-pressing method. The composite was tested within the frequency range of K-band where the shielding effectiveness was achieved as 30 dB while using the thin films of 0.25 mm thickness. Barium strontium titanate (BST) was synthesized with RGO and Fe_3_O_4_ with the addition of polypyrrole polymer via chemical oxidative polymerization. The testing was made within the X-band frequency range where the shielding effectiveness of 48 dB was achieved [148]. Using the Hummers method, RGO and polyetherimide (PEI) polymer were synthesized to form a shielding composite that was tested in the range of X-band frequency. With the RGO loading of 2.5 wt.%, the maximum shielding effectiveness of 26 dB was achieved [149].

Hong et al. [150] evaluated the anisotropic EMI shielding effectiveness of polymer-based composites. Magnetic responsive reduced graphene oxide (Fe_3_O_4_@RGO) as filler material was synthesized for controlling the orientation of reduced graphene oxide in thermoplastic polyurethane (TPU), where the magnetic field was applied to control the orientation of Fe_3_O_4_@RGO in in-plane and out-plane direction. A comparison was made between aligned Fe_3_O_4_@RGO/TPU, random Fe_3_O_4_@RGO/TPU and random RGO/TPU composites. Results revealed that the random Fe_3_O_4_@RGO/TPU composites shown an increase in EMI shielding effectiveness by 224% over random RGO/TPU composites. Whereas in-plane aligned Fe_3_O_4_@RGO showed 250% improved EMI shielding effectiveness over random RGO/TPU composites. The results proved that in determining the EMI shielding effectiveness, the orientation of fillers plays a vital role. Hu et al. [151] synthesized graphene sponge (G) with polyurethane to form a shielding composite. With a sample thickness of 9 mm and graphene loading 18.7 wt.% shielding effectiveness of 35 dB was achieved in the X-band frequency range. In another study, TPU was synthesized with thermally reduced graphene nanosheets (TRG) via the solution blending method and was tested for its shielding efficiency in Ku-band. The concentration of TRG was from 0 to 5.5 vol% where the maximum total shielding effectiveness was achieved as 32 dB at 5.5 vol% while keeping the sample thickness as 2 mm [152]. Zubair et al. [153] synthesized thermally reduced graphene oxide (TRGO) and barium hexaferrite (BaFe) with thermoplastic TPU via the solution casting method. While keeping the sample thickness as 0.25 mm, EMI shielding effectiveness of -61 dB was achieved at 12.5 GHz frequency.

Poly(3,4-ethylenedioxythiophene) (PEDOT) was synthesized with RGO and SrFe_12_O_19_ nanoparticles through in-situ growth. The EMI shielding composite was tested in the X-band range where the shielding effectiveness of 42.29 dB was achieved with 2.5 mm thickness and 62 dB with 4.66 mm thickness [154]. PEDOT and RGO were also synthesized with PbTiO3 via chemical oxidative polymerization where the shielding effectiveness of 51.94 dB was achieved within the frequency range of 12.4–18 GHz at 2.5 mm thickness [155]. In another study PEDOT:PSS was synthesized with Fe_3_O_4_ and RGO to form a shielding composite. The testing was made within the range of 2–18 GHz where the maximum reflection loss of −61.4 dB was achieved with 3.86 mm sample thickness [156]. Shukla [157] synthesized Fe_3_O_4_ with carbon (C) and polypyrrole (PPy) via hydrothermal and chemical oxidative polymerization to form a shielding composite. It was observed that by keeping the carbon loading up to 2 wt.% and PPy up to 8 wt.%, with the sample thickness 0.8 mm, shielding effectiveness > 28 dB was achieved at 2–8 GHz frequency range. In another study, polypyrrole was used with FeCo and RGO to form a shielding composite via a three-step method. The testing was made within the range of 2–18 GHz where the maximum reflection loss of −40.7 dB was attained at 4.5 GHz when the sample thickness was 2.5 mm [158]. Yan et al. [159] optimized three different polymer-based composites i.e., RGO-PANI-NiFe_2_O_4_, RGO-PPy-NiFe_2_O_4_ and RGO-PEDOT-NiFe_2_O_4_. With a material thickness of 2.4 mm, 1.7 mm and 2 mm, a reflection loss of −49.7 dB, −44.8 dB and −45.4 dB was achieved within the 2–18 GHz frequency range. It can be observed that the highest reflection loss was achieved by the PANI polymer composite.

Zuo et al. [160] synthesized polymethyl methacrylate (PMMA) with graphene and Li_0.35_Zn_0.3_Fe_2.35_O_4_ where the testing was made within the range of 2–18 GHz. A reflection loss of −46.1 dB was achieved with 4 mm thickness. Sharif et al. [161] optimized PMMA and RGO to form a shielding composite where the testing was made within the X-band. With 2.9 mm sample thickness and 2.6 vol% RGO, shielding effectiveness of 63.2 dB was achieved. Joseph et al. [162] synthesized two different polymer composites for EMI shielding. The first combination was of PMMA with graphene, whereas, the second combination was of polyvinyl chloride (PVC). The shielding effectiveness of 21 dB and 31 dB was achieved within the X-band frequency range with sample thickness as 2 mm and graphene loading as 20 wt.%.

Rao et al. [163] synthesized Fe_3_O_4_ with single-layer graphene-assembled porous carbon (SLGAPC) and polyvinyl alcohol (PVA) via the solution casting method. With a thickness of 0.3 mm, the shielding effectiveness of 20 dB was achieved in the X-band frequency range. Khodiri et al. [164] used PVA, graphene (Gr) and magnetite (Fe_3_O_4_) to form a shielding composite. With 0.2 mm thickness and little graphene loading of 0.08 wt.%, shielding effectiveness of 40.7 dB was achieved within the X-band frequency range. Li et al. [165] explored polyether-ether-ketone (PEEK) polymer with GNP and carbonized loofah fibre (CLF) to form a shielding composite. Keeping the testing within X-band, shielding effectiveness of 27.1 dB was achieved with 9 wt.% of CLF. Yadav et al. [166] used NiFe_2_O_4_, RGO and polypropylene to form a shielding composite. The testing was in the range of 6-8 GHz where high shielding effectiveness of 29.4 dB was achieved with 2 mm thickness and 5 wt.% RGO loading. Table 2 shows a summary of polymer-based composites.

## 6. Discussion

**Figure 8:**
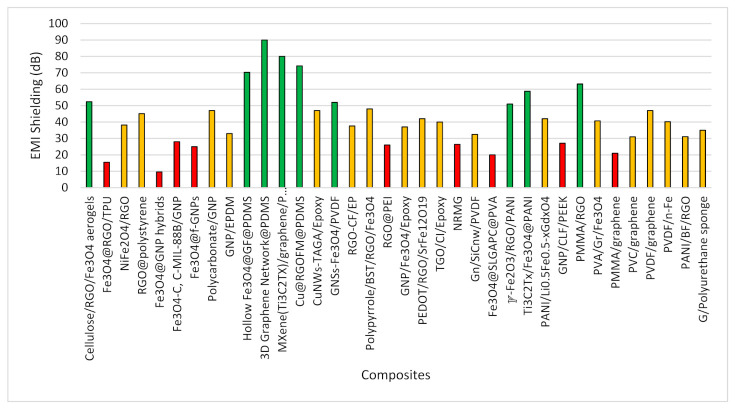
Composites shielding effectiveness within X-band range.

Exploring the articles, it was revealed that mostly graphene and iron-based composites were utilized within the X-band range. Later, the inclusion of polymers was tested within the same range. However, few studies trace the higher frequency ranges where the role of polymers cannot be disregarded. Figure 8 shows the shielding material composites which were targeted to deal with EMI within the X-band range. A clear understanding can be drawn that the highest shielding effectiveness was achieved by the addition of polymer composites. Whereas the combination of graphene and iron-based composites are not that beneficial to attain the targeted shielding effectiveness. Among polymers, PDMS, PANI, PMMA and PVDF showed remarkable results in the X-band frequency range. However, the role of PMMA needs to be investigated further as it also showed less shielding effectiveness with graphene. Among other polymers, less shielding effectiveness was also seen by PVA, PEI and TPU composites proving that their combinations need further exploration with other suitable materials.

**Figure 9:**
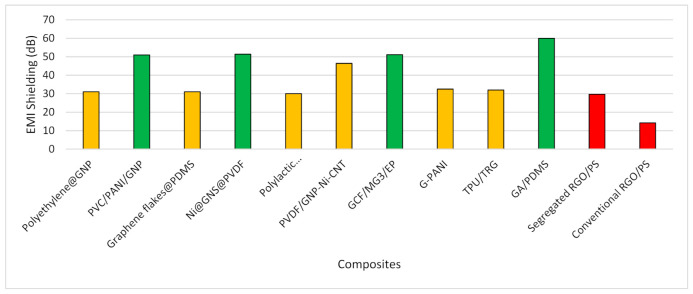
Composites shielding effectiveness higher than X-band range.

In a similar manner, composites were presented in Figure 9 which were utilized to observe the shielding effectiveness in frequency ranges greater than X-band. The higher shielding effectiveness was achieved with PDMS, Epoxy, PVDF and PANI. However, polystyrene (PS) polymer showed poor results in higher frequency. Interestingly, it was revealed that the combination of two polymers, i.e., PVC and PANI gave good shielding effectiveness, however, the inclusion of two polymers to form one single composite needs further investigation.

**Figure 10:**
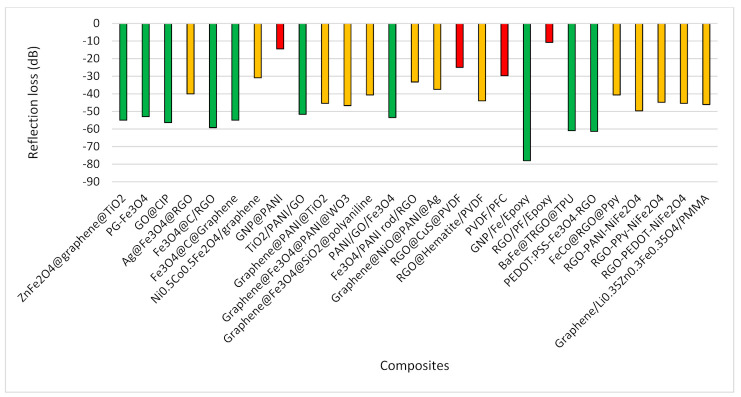
Reflection Loss.

In a few studies, researchers calculated the reflection loss only instead of providing total shielding effectiveness where the summary is provided in Figure 10. A higher reflection loss was observed in epoxy, PEDOT, TPU and PANI polymer composites. Interestingly, graphene and iron-based composites also showed a remarkable reflection loss. However, in a few studies, the inclusion of polymer composites showed less reflection loss.

Initially, metals were used as electromagnetic shielding materials to deal with electromagnetic inferences, however, studies showed that due to rigidness, ductile nature, heavyweight, and corrosion effect, they were not endorsed for additional utilization. Later, graphene emerged as a promising new material in the field of EMI where it showed remarkable results. Though individually it possesses less dielectric or magnetic properties yet, with the combination of iron and polymer composites, it showed excellent shielding effectiveness. Polymers, less explored, but effective, also showed significant results with a combination of graphene, where shielding effectiveness was achieved in a higher frequency range. However, there is a need for further exploration of graphene, iron-based polymer composites at higher frequencies as most of the researchers worked on X-band frequency range or slightly touch Ku-band. The graphene and iron base conductive polymer composites (CPC) provides maximum shielding effectiveness for a very short duration and decreases drastically with the increase in the X-band frequency range which is a problem and is very less explored with this family. The role of increasing thickness and loading cannot be ignored as in most of the studies, the shielding effectiveness was increased by increasing the filler content and thickness. However, there is a need to explore the new combinations to provide better shielding effectiveness in the higher frequency range. Moreover, it was observed that the combination of two polymers came up with better shielding performance yet requires further study. Those polymers which performed excellently in a higher frequency range can be combined by making an adequate synthesis. By doing so, the difficulties in achieving significant shielding effectiveness in a higher frequency range can be minimized.

## 7. Drawbacks and Future Direction

Various studies have been performed on EMI applications, but there are still a few limitations and drawbacks in the carried-out work which requires vital attention. Researchers have tried different methods to synthesize composites, however, adoption criteria for the different material synthesis methods is still missing in the literature. Although graphene is a significant material in composite formation, its dispersion is not easy. Similar is the case of polymers, where researchers usually did not properly quote the appropriate amount of solvent and curing agent which is required to mix polymers with graphene and iron. Besides that, in EMI applications, few researchers have calculated the reflection properties, few find the absorption and few have calculated the overall EMI shielding value.

Bringing the discussion into a net shell, researchers did not provide complete information about the composite synthesis process which makes it difficult to understand the role of different methods, amount of added solvent and curing agent, and the temperature effect during the whole composite-formation process. Considering the above statement, besides exploring the new combinations, there is a need to evaluate the comparison of methods for composites formation with their required parameters. Moreover, it needs to be stated that even in EMI shielding effectiveness which material performs better in absorption, and which performs better in reflection, as the composite applications may vary according to the influenced EMI working field. Moreover, new combinations of graphene-based polymers need further exploration in the higher frequency range as most of them currently dealing within the X-band frequency range.

## 8. Conclusions

Electromagnetic interferences have been studied for a long time and many composites have been developed to deal with this problem. A review was carried out in this study where previously conducted studies in electromagnetic inferences with the combination of Graphene@Iron, Graphene@Polymer, Iron@Polymer and Graphene@Iron@Polymer composites were examined. Graphene, iron, and polymers composites represent the most promising materials in EMI applications due to their unique properties. It was observed that the shielding effectiveness depends on the thickness and amount of the filler content, where their increasing proportions can enhance the effectiveness. Polymers show efficient performance with graphene and iron combinations where PDMS, PANI, PMMA and PVDF were effective in the X-band range. In the higher frequency range, PDMS, epoxy, PVDF and PANI polymers were astonishing in providing effective shielding. It was also observed that most of the studies were conducted in the X-band frequency range and few in the higher frequency range with the same composites’ family, so exploring new combinations within the same family to reach a higher frequency range is still a knowledge gap.

## Figures and Tables

**Table 1:** Summary of Graphene@Iron-based composites.

S. No	Material	Thickness	Loading	Methods	Frequency	Shielding Effectiveness	Year	Reference
1	ZnFe_2_O_4_@graphene@TiO_2_	2.5 mm	-	Hydrothermal method	3.8 GHz	−55 dB	2017	[81]
2	Cellulose/reduced graphene oxide (RGO)/Fe_3_O_4_ aerogels	0.5 mm	3 wt.%	Scalable method	8–12 GHz	49.4 dB	2020	[83]
		2 mm	8 wt.%			52.4 dB		
3	NiFe_2_O_4_/RGO	2 mm	-	Solvothermal method	10.8 GHz	38.2 dB	2020	[84]
4	MoS₂-RGO/CoFe₂O₄	1.4 mm	-	Hydrothermal method	8–12 GHz	19.26 dB	2019	[85]
5	Fe_3_O_4_@GNP hybrids	1 mm	-	1. Co-precipitation technique 2. Solvent-less approach	8–12 GHz	9.6 dB	2020	[86]
6	Fe_3_O_4_-C, C-MIL-88B/GNP	0.11 mm	-	Filtration-assisted self-assembly method	8–12 GHz	28 dB	2019	[87]
7	Fe_3_O_4_@f-GNPs	2 mm	-	Solvothermal method	12 GHz	25 dB	2020	[64]
8	PG-Fe_3_O_4_	6.1 mm	-	In-situ growth	5.4 GHz	−53 dB	2017	[88]
9	GO@CIP	1.9 mm	-	Wet stirring process	0–18 GHz	−56.4 dB	2019	[89]
10	Ag@Fe_3_O_4_@RGO	2 mm	-	Solvothermal method	2–18 GHz	−40.05 dB	2015	[90]
11	Fe_3_O_4_@C/RGO	3.57 mm	-	Solvothermal method	2–18 GHz	−59.23 dB	2020	[91]
12	NRMG	1.6 mm	-	Self-assembly method	8–12 GHz	26.4 dB	2018	[92]
13	Fe_3_O_4_@C@Graphene	1.5 mm	-	Hydrothermal method	2–18 GHz	−55.02 dB	2018	[92]
14	G-F	1.9 mm	-	Hydrothermal method	2–18 GHz	20 dB	2016	[93]
15	Ni_0.5_Co_0.5_Fe_2_O_4_/graphene	4 mm	-	Hydrothermal method	0.58–1.19 GHz	−30.92 dB	2018	[94]
16	RGO/CNF@Ag-Fe_3_O_4_	0.11 mm	-	Vacuum-assisted filtration method	8–12 GHz	21 dB	2020	[95]

**Table 2:** Summary of Polymer-based composites.

S. No	Material	Thickness	Loading	Methods	Frequency	Shielding Effectiveness	Year	Reference
1	PVC/PANI/GNP	-	5 wt.%	Solution processing method	18–20 GHz	51 dB	2019	[107]
2	GNP@PANI	1.5 mm	-	In-situ growth	12 GHz	−14.5 dB	2019	[108]
3	TiO_2_/PANI/GO	3.12 mm	-	In-situ growth	2–18 GHz	−51.7 dB	2017	[109]
4	Graphene@PANI@TiO_2_	1.5 mm	-	1. In-situ growth 2. Hydrothermal method	2–18 GHz	−45.4 dB	2016	[110]
5	Graphene@Fe_3_O_4_@PANI@WO_3_	4 mm	-	1. Hydrothermal method 2. Chemical oxidation polymerization	9.4 GHz	−46.7 dB	2017	[111]
6	Graphene@Fe_3_O_4_@SiO_2_@polyaniline	2.5 mm	-	Dilute polymerization	12.5 GHz	−40.7 dB	2015	[112]
7	PANI/GO/Fe_3_O_4_	3.91 mm	-	Hummers method	2–18 GHz	−53.5 dB	2015	[113]
8	Ag@Graphene/PANI	-	5 wt.%	In-situ growth	0.4–1.6 GHz	29.33 dB	2013	[114]
9	Fe_3_O_4_/PANI rod/RGO	3.5 mm	-	Facile method	2–18 GHz	−33.3 dB	2019	[115]
10	Graphene@NiO@PANI@Ag	3.5 mm	-	1. Hydrothermal method 2. In-situ growth	2–18 GHz	−37.5 dB	2017	[116]
11	G-PANI	1.5 mm	-	In-situ growth	2–18 GHz	32.5 dB	2017	[117]
12	ℽ-Fe_2_O_3_/RGO/PANI	2.5 mm	-	1. Chemical oxidation polymerization 2. In-situ growth	8–12 GHz	51 dB	2014	[118]
13	Ti_3_C_2_T_x_/Fe_3_O_4_@PANI	12.1 µm	-	Co-precipitation method	8–12 GHz	58.8 dB	2020	[119]
14	PANI/BF/RGO	-	-	Citrate precursor method	8–12 GHz	31.1 dB	2016	[120]
15	PANI/Li_0.5_Fe_0.5-x_Gd_x_O_4_	2 mm	-	In-situ growth	8–12 GHz	42 dB	2019	[121]
16	RGO@polystyrene	-	3.47 vol%	High-pressure solid-phase compression moulding	8–12 GHz	45.1 dB	2015	[99]
17	Segregated RGO/PS	2 mm	10 wt.%	Hot compressed method	0–20 GHz	29.7 dB	2018	[122]
	Conventional RGO/PS					14.2 dB		
18	Polycarbonate/GNP	1 mm	-	Facile solution method	8–12 GHz	35 dB	2018	[123]
		2 mm	-			47 dB		
19	Polyethylene@GNP	-	15.6 vol%	Injection moulding process	18 and 26.5 GHz	16 dB	2018	[124]
			19 vol%			31.6 dB		
			3 wt.%			12 dB		
			10 wt.%			31 dB		
20	GNP/EPDM	0.3 mm	8 wt.%	Ultrasonication technique	8–12 GHz	33 dB	2019	[125]
					12.4–18 GHz	35 dB		
21	Hollow Fe_3_O_4_@GF@PDMS	-	4 wt.%	Solvothermal method	8–12 GHz	45 dB	2020	[129]
			8 wt.%			65 dB		
			12 wt.%			70.3 dB		
22	3D Graphene Network@PDMS	0.25 mm	1.2 wt.%	Chemical vapor deposition	8–12 GHz	40 dB	2020	[129]
		0.75 mm				90 dB		
23	MXene(Ti_3_C_2_T_X_)/graphene/PDMS	1 mm	-	Chemical vapor deposition	8–12 GHz	80 dB	2020	[130]
					26.5–40 GHz	77 dB		
24	Graphene flakes@PDMS	-	0.1 wt.%	Mechanical mixing	0.6 THz	6.5 dB	2018	[126]
			3 wt.%			12 dB		
			10 wt.%			31 dB		
25	Cu@RGOFM@PDMS	0.5 mm	-	Hummers method	8–12 GHz	74.2 dB	2020	[127]
26	GA/PDMS	2.5 mm	-	1. Ultrasonication technique 2. Hydrothermal method	2–18 GHz	60 dB	2020	[128]
27	Ni@GNS@PVDF	0.5 mm	-	Ultrasonication technique	18–26 GHz	43.3 dB	2020	[132]
		0.7 mm				51.4 dB		
28	RGO@CuS@PVDF	1 mm	-	Hydrothermal method	12–18 GHz	−25 dB	2020	[133]
29	GNSs-Fe_3_O_4_/PVDF	0.3 mm	-	Facile layer-by-layer coating	8–12 GHz	52 dB	2020	[134]
30	RGO@Hematite/PVDF	-	5 wt.%	In-situ growth	2–18 GHz	−43.97 dB	2014	[135]
31	Gn/SiCnw/PVDF	1.2 mm	-	1. Electrostatic assembly 2. Solution casting method	8–12 GHz	32.5 dB	2020	[136]
32	PVDF/graphene	20 µm	15 wt.%	Solution casting method	8–12 GHz	47 dB	2018	[137]
33	PVDF/GNP-Ni-CNT	0.6 mm	-	Solvent casting method	12–18 GHz	46.4 dB	2020	[138]
34	PVDF/n-Fe	1.93 mm	-	Hot-moulding process	12–18 GHz	40.21 dB	2016	[139]
35	PVDF/PFC	2 mm	1 wt.%	Solution blending process	12–18 GHz	−29.7 dB	2017	[140]
36	CuNWs-TAGA/Epoxy	-	7.2 wt.%	Thermal annealing method	8–12 GHz	47 dB	2020	[132]
37	RGO-CF/EP	3–5 mm	-	1. Electrophoretic deposition 2. Chemical reduction	8–12 GHz	37.6 dB	2016	[141]
38	GNP/Fe_3_O_4_/Epoxy	-	7 wt.%	Co-precipitation method	8–12 GHz	37.03 dB	2019	[142]
39	GNP/Fe/Epoxy	5 mm	5 wt.%	Sonication method	1–65 GHz	−78 dB	2020	[143]
40	TGO/CI/Epoxy	4 mm	-	Centrifugal mixing method	8–12 GHz	40 dB	2015	[144]
41	GCF/MG_3_/EP	-	0.5 wt.%, 9 wt.%	Hummers Method	18–26 GHz	51.1 dB	2017	[145]
42	RGO/PF/Epoxy	3 mm	60 wt.%	Solution mixing method	2–18 GHz	−10.26 dB	2020	[146]
43	Polylactic acid/Biochar/Graphite	0.25 mm	-	Hot-pressing method	18–26.5 GHz	30 dB	2019	[147]
44	Polypyrrole/BST/RGO/Fe_3_O_4_	22.8 × 10.03 × 2.5 mm	-	Chemical oxidative polymerization	8–12 GHz	48 dB	2018	[148]
45	RGO@PEI	-	2.5 wt.%	Hummers Method	8–12 GHz	26 dB	2018	[149]
46	Fe_3_O_4_@RGO/TPU	1 mm	-	Solution casting method	8–12 GHz	~15.51 ± 1.6 dB	2020	[150]
47	G/Polyurethane sponge	9 mm	18.7 wt.%	Hydrothermal method	8–12 GHz	35 dB	2019	[151]
48	TPU/TRG	2 mm	5.5 vol%	Solution blending method	12–18 GHz	32 dB	2017	[152]
49	BaFe@TRGO@TPU	0.25 mm	-	Solution casting method	0.1–20 GHz	−61 dB	2020	[153]
50	PEDOT/RGO/SrFe_12_O_19_	2.5 mm	-	In-situ growth	8–12 GHz	42.29 dB	2019	[154]
		4.66 mm				62 dB		
51	PEDOT/RGO/PbTiO_3_	2.5 mm	-	Chemical oxidative polymerization	12.4–18 GHz	51.94 dB	2018	[155]
52	PEDOT:PSS-Fe_3_O_4_-RGO	3.86 mm	-	Hydrothermal method	2–18 GHz	−61.4 dB	2018	[156]
53	Fe_3_O_4_/C:PPy	0.8 mm	2.8 wt.%	1. Hydrothermal method 2. Chemical oxidative polymerization	2–8 GHz	>28 dB	2019	[157]
54	FeCo@RGO@PPy	2.5 mm	-	1. Hydrothermal method 2. In-situ growth	2–18 GHz	−40.7 dB	2017	[158]
55	RGO-PANI-NiFe_2_O_4_	2.4 mm	-	1. Hummers method 2. Solvothermal method	2–18 GHz	−49.7 dB	2016	[159]
	RGO-PPy-NiFe_2_O_4_	1.7 mm				−44.8 dB		
	RGO-PEDOT-NiFe_2_O_4_	2 mm				−45.4 dB		
56	Graphene/Li_0.35_Zn_0.3_Fe_0.35_O_4_/PMMA	4 mm	-	3D printing method	2–18 GHz	−46.1 dB	2020	[160]
57	PMMA/RGO	2.9 mm	2.6 vol%	Self-assembly technique	8–12 GHz	63.2 dB	2017	[161]
58	PMMA/graphene	2 mm	20 wt.%	Hot compression method	8–12 GHz	21 dB	2019	[162]
	PVC/graphene					31 dB		
59	Fe_3_O_4_@SLGAPC@PVA	0.3 mm	-	Solution casting method	8–12 GHz	20 dB	2015	[163]
60	PVA/Gr/Fe_3_O_4_	0.2 mm	0.08 wt.%	Hummers method	8–12 GHz	40.7 dB	2020	[164]
61	GNP/CLF/PEEK	-	9 wt.%	Compression moulding method	8–12 GHz	27.1 dB	2019	[165]
62	NiFe_2_O_4_-RGO-Polypropylene	2 mm	5 wt.%	1. Hummers method 2. Hot press method	6–8 GHz	29.4 dB	2019	[166]

## Data Availability

All data is available within the manuscript.
